# Evaluation of the application opportunities of precision livestock farming (PLF) for water buffalo (*Bubalus bubalis*) breeding: SWOT analysis

**DOI:** 10.5194/aab-66-41-2023

**Published:** 2023-01-27

**Authors:** Orhan Ermetin

**Affiliations:** Department of Animal Science, Faculty of Agriculture, Yozgat Bozok University, 66100, Yozgat, Türkiye

## Abstract

The use of technology in agriculture is increasing daily with the development of technology in all areas. With the help of PLF (precision livestock farming) technologies and efficient use of inputs, economic, environmentally friendly, and better-quality products are obtained. Significantly its use in dairy cattle is increasing daily, contributing to sustainable milk production in both economic and ecological terms. As the demand increased in the world for water buffalo meat, milk, and dairy products, different breeding systems have been applied for more and higher-quality production purposes. This way the number of water buffalo farms breeding in intensive conditions is increasing. It is necessary to investigate the possibilities of using PLF technologies, which are still widespread in dairy cattle, in water buffalo breeding, and to benefit from the advanced technology in this regard. This study aims to discuss the applicability of PLF technologies by surveying buffalo breeders. With the data obtained from the survey results made with the water buffalo breeders, the strengths, opportunities, threats, and effects of the weaknesses were discussed with the SWOT analysis.

## 1 Introduction

The term “precision agriculture” has been applied in various areas of agriculture for the past 30 years, and the basic principles of precision agriculture are applied to livestock systems called PLF (precision livestock farming). Briefly, PLF is an approach to managing livestock systems by reducing workforces and increasing productivity, economic viability, efficiency, sustainability, and welfare (Banhazi et al., 2012; Berckmans, 2017; Wishart, 2019). PLF technologies also enable breeders to detect and control the health and well-being of animals at any time with the help of continuous, direct monitoring or observation of animals. In this way, there will undoubtedly be increases in the efficiency and quality of products of healthy and “prosperous” animals in the long term (Berckmans, 2014).

Using technology, the physiological, behavioral, and production indicators of animals can be measured. Problems that people do not notice are noticed thanks to sensors, cameras, and activity meters. This way, breeders can focus on the health and performance of each animal. Using PLF information and technology to improve animal health, animal welfare, and farm efficiency is now an area of scientific and commercial interest. Using technology, PLF technology systems adopt sustainable animal production based on animal health, both in economic and ecological terms. The main targets are improving a farm's economic, social, and environmental performance, making timely and conscious decisions, and reducing drug use with preventive health practices. Hostiou et al. (2017) reported that the time-saving feature was one of the most important reasons companies select PLF technologies. Feeding and milking animals are the most time-consuming activities on dairy farms (Hostiou et al., 2017). However, obtaining the expected benefits from these systems is possible with the knowledge of the functions and effective use of relevant systems (Göncü and Gökçe, 2017).

The applications of research and technological advances in the world in farm animal breeding have started the development of PLF farming. With this developing technology, sensors (cameras, microphones, and accelerometers) are used to prepare algorithms to detect the welfare of animals without disturbing them with sounds and movements and to predict productivity (Greenwood et al., 2014). PLF technologies enable automatic remote sensing and monitoring of images, sounds, tracking data, weight, and biological measurements for early detection of diseases, physiological conditions, and the well-being of animals using real-time analytics.

All kinds of activities and productions important for animal behavior and welfare can be monitored and evaluated by image analysis with 2D or 3D cameras and tracking systems installed in farms (Pezzuolo et al., 2018). By doing this, the life cycle of animals is recorded, and incorrect practices can be intervened immediately. With the help of PLF technologies, fast and error-free access is achieved to the records in businesses, and these records can be accessed from anywhere using web technologies (Borchers et al., 2015).

Many genetic, feeding, and cultivation studies are conducted about water buffaloes, which produce the most milk after cattle in the world. However, there are few studies on water buffalo breeding in intensive conditions and compliance with PLF technologies. More studies are needed on water buffalos' need to benefit more from PLF technologies in reproductive problems (hidden rut, non-fertilization, etc.), heat stress, and milking. While PLF technologies are applied in farms, a certain time is required for water buffalo breeding. The most critical issues are recognizing the temperament of the buffalo, training the breeders, and adapting the PLF technologies used in dairy cattle to buffalo breeding.

## 2 Water buffalo breeding in the world

Water buffalo (*Bubalus bubalis*), a farm animal with significant economic production in the world, is used in areas such as meat, milk, bollard pull, leather, horn, and manure. Two subspecies represent the buffalo: swamp and river, with diploid chromosome numbers of 48 and 50, respectively (Harisah et al., 1989). Approximately 97 % of 200 million water buffaloes in the world live in Asia, 2.04 % in Africa, and about 1 % in South America, Australia, and Europe (FAOSTAT, 2020). The distribution of the number of buffaloes in the world in 20 countries with the largest number of buffaloes is given in Table 1.

**Table 1:** Top 20 countries with the most water buffalo in the world (FAOSTAT, 2020).

Countries	Number of water buffalo (head)
India	109 719 011
Pakistan	41 191 000
China	27 223 427
Nepal	5 257 591
Myanmar	4 125 140
Philippines	2 865 715
Vietnam	2 332 754
Egypt	1 671 378
Brazil	1 502 482
Bangladesh	1 493 000
Laos	1 234 000
Indonesia	1 179 342
Thailand	923 533
Cambodia	639 922
Italy	407 030
Colombia	338 567
Sri Lanka	323 000
Iraq	233 453
Türkiye	192 489
Iran	171 156
World sum	203 535 363

Most water buffaloes bred for milk are in India, Pakistan, China, Nepal, and Myanmar (Borghese and Mazzi, 2005; Deb et al., 2016). In many parts of the world, especially in Southeast Asian countries, swamp buffalo provide 20 %–30 % of draft power and also provide meat (Mintoo et al., 2019). In other regions, such as India, river buffalo are primarily kept for milk production and secondarily for meat production (Safari et al., 2018; De la Cruz-Cruz, 2019). Water buffalo, which is usually bred in extensive conditions, has many advantages like converting poor-quality roughage into milk and meat, higher endurance compared to other farm animals against diseases, cholesterol in milk, and low cholesterol and fat content in the meat. The average milk yield of buffaloes in some countries is 1934 kg per animal per year in Pakistan, 1880 kg per animal per year in India, 1000 kg per animal per year in Vietnam, 867 kg per animal per year in Nepal, 508 kg per animal per year in Sri Lanka, 563 kg per animal per year in China, 410 kg per animal per year in Bangladesh, and 815.9 kg per animal per year in Italy (Siddiky and Faruque, 2017).

Although water buffalo has not been valued much when compared to other farm animals until today, it has been focused strongly on in many countries because of its various characteristics and yield. In countries where there are suitable conditions, the necessary importance has been given to water buffalo breeding (Kul et al., 2018). Among the characteristics that give water buffalo this importance, there is endurance against nature conditions and diseases, high power in benefiting from feed, converting poor-quality roughage into meat and milk, cultivation costs being lower than cattle, and feeding is cost-effective (Atasever and Erdem, 2008).

In South and Southeast Asia, where most of the buffalo population lives in the world, the possibility of developing water buffalo production as a whole is associated with the use and implementation of scientific advances and related technologies in some key areas. Approximately 99.9 % of the buffalo presence in China is native swamp races, which have low milk and meat yield and are often used as bollard pull. However, in recent years, interest in water buffalo milk has increased, and the dairy industry has been developing (Yang et al., 2013). In South Asian Countries, the bollard pull of swamp buffaloes is used extensively in the cultivation of rice, corn, sugar cane, and coconut. River buffaloes, on the other hand, cover the family milk needs in rural areas, and commercial dairy businesses are observed near big cities (Cruz, 2007; Deb et al., 2016).

In a study conducted in Bangladesh, Rahman et al. (2019) determined the average milk production for the first, second, and third stages as 6.80, 4.30, and 2.00 L d ${}^{-1}$ . They also reported that milk yield in buffaloes increased after breeding in full intensive conditions as 11, 6.5, and 2.5 L d ${}^{-1}$ , respectively. In another study conducted in coastal areas, the same authors reported that applying technological advances to improve milk yield and quality in buffaloes bred in semi-intensive systems would contribute to national milk production.

Water buffalo breeders are marketing high-priced milk and dairy products in Italy with high genetic-value animals, effective water buffalo breeding, selection, recording procedures, and dissemination of artificial insemination. The annual milk yield in buffaloes is over 2200 kg . The average herd size of the water buffaloes bred for milk yield is 161.3 in free-stop stables in intensive conditions, and machine milking is performed twice a day. Each year, a total of 36 000 t of mozzarella cheese worth EUR 500 million is produced in Italy, 82 % of which is consumed in Italy and 18 % exported (Borghese, 2013).

Buffaloes were used for bollard pull in Central and South American countries; however, today, they cover the increasing demands for milk and meat production. It is an alternative-free source of production and income for small- and medium-sized enterprises (Zava, 2013). Similarly, Soysal (2013) reported that water buffaloes were bred in extensive conditions for family needs for milk in rural areas in Türkiye, and large and intensive farms were usually located near large cities.

## 3 PLF (precision livestock farming) technology and its importance

The term “precision livestock farming” was first used by Berckmans in 2004, and the researcher described PLF as “innovative implementations including measurements, estimates and data analysis of key variables to collect and analyze data from farm animals in a continuous and fully automated way”. In addition, the researcher also argued that the target of PLF was to “manage individual animals through continuous real-time monitoring of health, welfare, efficiency, reproduction, and environmental impacts” (Berckmans, 2004, 2006, 2017).

PLF technologies ensure nutrition, the ability to collect data like regular milk registration (efficiency and components), pedometers, pressure plates, milk conductivity indicators, automatic rut detection, body weight, temperature, bedtime, ruminal pH, heart rate, nutritional behavior, blood tests, respiratory rates, rumination times, scoring movement ability using image analysis at individual animal level. In this way, a focus is made on disease prevention, health, and performance, minimizing drugs to be used (Yıldız and Özgüven, 2018). PLF can be defined as the management of livestock production by using transaction engineering principles and technology (Morgan-Davies et al., 2018).

The purpose of PLF is to manage individual animals with real-time continuous monitoring of health, welfare, production/reproduction, and environmental effects. The term “continuous” means that PLF technology constantly measures and analyses (Berckmans, 2015). Breeders receive a warning from the PLF system when something goes wrong to attend to animals that need to be considered at that moment. Monitoring can be performed with cameras and real-time image analysis, microphones and real-time audio analysis, or sensors around or above animals (Berckmans, 2017). One of the goals of PLF is to make animal breeding more economical, socially, and environmentally sustainable (Vranken and Berckmans, 2017).

Innovative systems based on wearable (attached to animals) sensors (pedometers, nutritional behavior activity meters, etc.) and automated calculation procedures have been adopted recently to improve animal health and welfare and ensure effective and accurate monitoring and analysis of animal behaviors. Other low-cost monitoring systems are based on wearable sensors and other devices (e.g., computers and wireless networks used increasingly because of easy integration; Mancino, 2016). Schewe and Stuart (2015) reported that sensors reduced the pressure, mental workforce, and stress conditions of breeders by regularly producing records and information. Wishart (2019) said that PLF practices had four basic principles regardless of animal species and production purposes: using scientific information about the system, measurement/monitoring/management of the system, proper technology use, and determining the variation in the system. PLF can be used to monitor the physical environment of animal buildings, including animal growth and behaviors, product efficiency, endemic diseases, and micro-environmental and gas pollutant emissions (Fournel et al., 2017). PLF technologies allow intervention as soon as the first signs of impaired welfare or pathogens emerge (Tullo et al., 2019).

It must be noted that the development of PLF systems requires cooperation among various disciplines (Carpentier et al., 2019). For example, a critical value (gold standard) must be set by detecting the amount of cough for a certain period of time from an audio signal that is measured continuously by sound sensors for the development of a system for detecting respiratory infections based on this value. Then, based on the intensity of the detections from critical values, a series of predefined features (i.e., the exact start and endpoint of the cough) are determined from audio and video recordings. For this reason, PLF methodology requires high-level cooperation among many study areas, including animal scientists (e.g., physiologists, behavioral scientists, and nutritionists), laboratory technicians, software developers, and engineers (Norton et al., 2019).

PLF techniques can minimize the adverse environmental effects of farms, improve economic sustainability and welfare of animals and employees (at least in some criteria), and minimize the spread of diseases which might spread to humans (Werkheiser, 2018).

For the technology to be used in PLF farming to be considered the ideal technology, it must have the following characteristics:

- It should explain the underlying biological process.
- It can be translated into meaningful action.
- It should have a low cost.
- It should be flexible, solid, and reliable.
- Information should be easily accessible to the farmer.
- The farmer should be involved in the development of technology – not only as a tester but as a developer in the development of all stages.
- Commercial demonstrations should exist.
- It should have a continuous effect and feedback loop (Bewley et al., 2015).

The success of PLF in a livestock business depends on many factors. It should be considered that the system might have positive or negative effects on the business (Jago et al., 2013). Although it has benefits like saving workforce, standardization, animal health, welfare protection, etc., the technology requires additional responsibilities like the needs/costs, close recognition of animals and their characteristics, the use of data storage, staff training, etc. However, the lack of clear cost–benefit data on PLF technologies is one of the most important limiting factors for commercializing PLF technologies (Kamphuis et al., 2015).

## 4 Behavioral characteristics of water buffaloes and compliance with technology in dairy enterprises

Water buffaloes (*Bubalus bubalis*) are more timid or aggressive than cattle. These characteristics also vary between races and within the herd. De Lima Carvalhal et al. (2017), who investigated the relationship between milk yield and quality characteristics of buffalo temperament, determined lower fat contents and a lower number of somatic cells in more reactive water buffaloes. Soysal (2009) reported that buffaloes were highly docile in 18 experiments in India, measuring a comparative temperament in Murrah buffaloes, hybrid dairy cows, and Red Sindhi cows. In this trial, 50 % of Murrah buffaloes were found to be docile, and 7 % were aggressive. (The rest of the water buffaloes were found to be limited or non-docile.) Opposite results are also reported. The important thing here is that the temperament scores were determined to decrease as the lactate sequence increased. Buffaloes are trainable animals (Nayak and Mishra, 1984). In other words, when they are in wild or wild conditions, they easily adapt to new conditions when trained in the direction of tamed behavior when they are aggressive. This proves that many breeders easily adopt machine-milking, contrary to what many breeders believe. It was shown that the temperament on milking affected the temperament in feed intake. In this respect, docile buffaloes are milked more easily than aggressive water buffaloes, managed more easily, and can be milked for longer durations by lowering milk in a shorter time, with more milk yield, with higher fat ratio, and faster intake of feed (Nayak and Mishra, 1984; Yılmaz, 2013). The herd and maternity instincts of water buffaloes are excellent. They take care of other calves and try to protect them from dangers. If they are made to become used to it, they can feed four calves at the same time. Although it is difficult to milk buffalos that give first births at the beginning of the lactation because they are not used to it and tend to hide their milk from their calves; however, they soon become used to it. Then, they are easily milked by hand or machine, as long as their accustomed carers milk them. Making them used to machine milking from an early age prevents breast elongation that can be observed in milking by hand. Machine milking is widely used in India, Pakistan, Egypt, Bulgaria, the Philippines, and Italy (Ermetin, 2021).

Water buffaloes show two basic behaviors (feeding/rumination and resting) in extensive conditions. Especially during the hot season, entering the water and wallowing in the mud represents certain water buffalo behaviors for adjusting the body temperature balance and external parasite protection. However, since they cannot show these behaviors in intensive conditions, it causes unwanted behaviors like excessive aggressiveness and sucking in buffaloes (Napolitano et al., 2013).

Monitoring water buffaloes with heat sensors are recommended, mainly to protect them from hot stress on farms. Thanks to monitoring and automatic participation systems, data can be collected for both breeders and consumers by making animal welfare plans for water buffaloes (De Rosa et al., 2009). Buffaloes exposed to stress during milking are more affined and do not descend milk under similar stress conditions than dairy cows. Some growers often use the hormone exogenous oxytocin to ensure that milk is completely drained from the breast (Cavallina et al., 2008). It is reported that significant improvements will be made in the cultivation and milk yields by making significant improvements in activities such as shelter, nutrition, reproduction, and milking in water buffalo cultivation (Safari et al., 2018). Seyfi (2013), when designing water buffalo stables, noted that instead of high-cost investments, new barn designs should be facilities that are affordable, take animal welfare and comfort into consideration, and facilitate milking.

**Table 2:** Number of sample enterprises by enterprise size groups

Size groups of	Number of enterprises	Number of enterprises
enterprises (head)	in the mainframe	in the sample
10–20 (small)	45	21
20–50 (middle)	30	14
≥ 51 (large)	9	5
Total	84	40

Water buffaloes do not pose difficulty in shipping and handling for the carers to whom they are accustomed in meadows or stables. They are frightened of unfamiliar strangers other than their own carer and are challenged to manage and milking. Since buffaloes have more herd instinct, they want to graze in groups. It is not easy to manage individual animals separated from the herd. They do not move far away from each other when grazing and consume feed slowly and for a long time (Ermetin, 2020). They often do not change the grazing area as long as they find adequate grass. When tied up in a stable for a long time, they cut each other's hair, leaving their entire bodies hairless. In females, the symptoms of anger are not as evident as in cattle. The desire to mate with water buffalo bulls is not as violent as cattle bulls. However, they easily distinguish even the water buffaloes that do not express their anger by checking the females in the herd at regular intervals. The individuals that are new in the herd struggle to surpass others. They are obedient to their carers and let them touch themselves (Soysal, 2009). When they are hungry and dehydrated, they approach their carers or make specific noises by raising their heads together. The water buffaloes that give birth do not let anyone near their baby except their carers. They are not picky about feeds (Kul et al., 2016). They prefer and eat roughage like reeds, straw, dry grass, alfalfa, and vetch. They also eat silage roughage without problems. They are resistant to sudden feed changes and hoof-and-mouth disease. Symptoms are not as severe as in cattle. They resist diseases that are caused by blood parasites (protozoon diseases). Many water buffaloes are often docile, can be hybridized among themselves, and are often guided even by a child (Atasever and Erdem, 2008; Yılmaz, 2013).

## 5 Material and methods

There are 4133 water buffaloes in total in 260 farms in Yozgat (TUIK, 2021). The study analyzed the results of face-to-face interviews with randomly selected 40 farm owners with 10 or more buffaloes. The enterprises that make up the sample give us a confidence level of 95 %–98 %, with an acceptable error rate of ± 10 %. In the study, the general situations of the enterprises were revealed, and the opportunities and threats waiting for the enterprises, the weaknesses, and strengths of the enterprises were revealed with the SWOT analysis (strength, weakness, opportunity, and threat analysis). In light of the SWOT analysis results, future planning and strategy suggestions were made for the sector.

Farms with fewer than 10 water buffaloes were excluded from the analysis due to the frequency distribution of the primary population. The enterprises with fewer than 10 water buffaloes are generally non-sustainable farms that also co-breed cattle. Since there are few enterprises with more than 50 heads, the study was examined in three layers formed such as between 10–20 (small), between 20–50 (middle), and more than 50 heads (large) (Table 2). The stratified random sample method was used to determine the number of farmers included in the sampling from the primary population (Yamane, 1967).

**Table 3:** PLF technology survey questions to evaluate in the SWOT analysis.

	10–20		20–50		≥ 51	
	(small)		(middle)		(large)	
	n	%	n	%	n	%
I'm informed about PLF technologies.	10	47.61	8	57.14	3	60
I think water resources are depleting and global warming is getting worse.	19	90.47	12	85.71	5	100
I want to invest in PLF technologies.	1	4.76	2	14.28	1	20
I think that the use of technology will increase the efficiency.	7	33.33	9	64.28	5	100
I'm receptive to technical advancements.	12	57.1	10	71.42	4	80
I want to continue water buffalo breeding in the future.	15	71.42	10	71.42	5	100
I don't have laboring problems.	12	57.14	11	78.57	2	40
I don't think PLF technologies convenient for water buffalo breeding.	18	85.71	8	57.14	1	20
I don't think investing in technology is essential since water buffaloes give little milk.	18	85.71	7	50	1	20
I use traditional methods to breed water buffaloes.	21	100	12	85.71	2	40
If I can sell milk and dairy products of water buffaloes at a reasonable price, I'll consider investing in technology.	8	38.09	5	35.71	3	60
If I can sell milk and dairy products of water buffaloes at a reasonable price, I'll consider increasing c number of water buffaloes on my farm.	14	66.66	10	71.42	3	60
If I were young, I'd invest in technology in water buffalo breeding.	5	23.80	3	21.42	2	40
If feed prices increase, even more, I may leave water buffalo breeding.	19	90.47	10	71.42	4	80
If I don't have marketing issues, I can put more budget into technology investments.	5	23.80	4	28.57	4	80
Government support should be increased.	21	100	14	100	5	100
If the government supports, I can establish PLF technology applications.	6	28.57	6	42.85	3	
Improvement studies should be increased in water buffalo breeding.	9	42.85	11	78.57	5	100
How long have you been breeding water buffaloes?						
1–5 years	3	14.28	0	0	0	0
5–10 years	2	9.52	1	7.14	0	0
10–20 years	6	28.57	2	14.28	1	20
More than 20	10	47.61	11	78.57	4	80

SWOT analysis is a technique used to identify the strengths, weaknesses, opportunities, and threats posed by the external environment of enterprises, organizations, techniques, processes, or situations. With the application of this technique, plans and strategies can be developed that will maximize the existing strengths and opportunities and minimize the effects of threats and weaknesses, considering internal and external factors (Seki and Biler, 2016). Survey questions for SWOT analysis are given in Table 3.

## 6 SWOT analysis of the usage potential of PLF technologies in water buffalo livestock

The strong sides, weak sides, opportunities, and threats in terms of SWOT analysis of the potential of using modern technologies and PLF technologies in water buffalo livestock farming are detailed below.

### Strong sides

The strong sides in SWOT analysis are used to reveal the success of enterprises and indicate their advantages. This study reflects the benefits and applicability of PLF technologies to water buffalo breeding. The benefits that will be brought by the use of technology in water buffalo breeding are the following:

- increasing yields (more milk/meat/calf yield),
- opportunity for individual care and feeding,
- reducing costs,
- replacement of human labor by machine power (less human labor),
- improving product quality,
- minimizing negative environmental impacts,
- improving animal health and welfare,
- opportunity to detect diseases in advance and proper intervention,
- rapid progress in improvement works,
- risk analysis and management (avoiding dangerous tasks),
- opportunity for monitoring,
- being more objective (less prejudice and impact on the breeder),
- creating a databank,
- water buffalo meat and milk products have high market value.

Reaching the instant information of all animals on the farm and using this information in decisions are very important in preventing adverse events and losses and for immediate interventions. In addition, based on these records, profitability and sustainability calculations can be made, which is very important in this respect. In addition, it is an advantage that herd management programs are flexible, can easily expand, and work in line with other automation systems. Another advantage is that the reports used here are broad and flexible based on user demands and possible future developments.

### Weak sides

The weak sides reveal the issues that businesses are missing. It reflects what challenges the applications of PLF technologies can pose to buffalo farming. The weak sides of the sector are the biggest obstacles to its development of the industry. In this context, strengthening the weaknesses is very important for the sector. The weaknesses are as follows:

- technology investment cost,
- technical knowledge deficiency (business owner, caretaker, technical staff),
- unconscious breeding techniques,
- requirement for effort and skill,
- inadequate size of business (animal count in the herd not being economic to use the technology),
- traditional techniques (water buffalo breeding generally being carried out under extensive conditions),
- difficulties in familiarizing with intensive conditions,
- temperament of water buffalo (timidity, aggressiveness, being used to machine milking, etc.),
- having horns,
- need for cooling, especially in hot weather conditions (having a bath in water, wallowing),
- technologic insufficiency,
- being dependent on factory feed,
- lack of examples at the breeder level,
- applicable and achievable resource problems,
- lower milk yield of water buffalo compared to cattle,
- need for high-yield breeding animals.

Water buffaloes are usually bred with minimal care and feeding in extensive conditions, and they must be made to become accustomed to intensive conditions. Breeders tend to avoid spending much effort and expense on breeding water buffaloes. When weaknesses are considered, it is seen that a large part of the problems can be dealt with good training and with a change in the breeding system. In addition, an increase in efficiency will contribute to the progress of the sector when technological advances are transferred to production.

### Opportunities

The opportunities are essential for the development of the business. It is the extent to which new developments in your sector are followed and the opportunities to turn them into opportunities. These are the possibilities that PLF technologies applications can create for water buffalo farming. Opportunities are the production systems and environmental factors that a business can turn into an advantage to become competitive in the sector. These factors are as follows:

- rapid progress and dissemination in technology,
- expansion and fall of costs in PLF technologies in livestock farming (decrease in the cost of investment),
- increasing demand for water buffalo milk and dairy products (especially in metropolitan cities),
- requirement for quality and standard product (meat/milk demand),
- having the brand value of water buffalo milk and meat products,
- buffalo products are valuable (sales at a high price compared to cattle products),
- breeder animal needs,
- labor force savings,
- grants and support,
- increasing social awareness of animal welfare,
- increasing scientific studies,
- good adaptations of water buffaloes.

The most remarkable among these opportunities is that they will become easier and cheaper to apply in water buffalo breeding with rapid progress and widespread technology. In this way, high-quality efficiency will be achieved with lower costs.

### Threats

Threats analyze the risks and obstacles that businesses may face. It explains the risks and dangers of PLF technologies applications in buffalo breeding. Threats are the negative aspects developing in favor of the sector. Taking measures to minimize these harmful aspects and developing policies and strategies are important for the sector's sustainability. When the adoption of PLF technologies in water buffalo breeding is considered, it is seen that progress is slow. These factors are as follows:

- global warming and drought,
- continuing the form of breeding for family needs (low cost),
- increase in feed prices,
- inadequate livestock policies,
- inadequate support,
- decreasing rural population (migration to metropolitan cities),
- young population not being interested in breeding (elderly people stay away from technology),
- cost–benefit rates are not at desired levels,
- not recognizing available technologies, fear of technology,
- high research and investment costs,
- previous bad experiences with technology,
- degree of the impact of resources used in breeding operations,
- management level that is necessary for the implementation of technology,
- risk carried by technology,
- difficulties in learning and using technology.

The most significant threat element observed seems to be the adaptation of technology, global warming, and difficulty implementing the intensive breeding system in water buffalo breeding.

## 7 Results and suggestions

In our present day, fast progress in technological development and its use in livestock have led to more precise and powerful technological means being accessible at more affordable prices. PLF technologies now constitute dynamic research and innovation industry and are renewed daily. PLF may become the key to sustainable livestock in the future. Using these technologies in water buffalo breeding, PLF will advance, and breeders will adopt the value of technology. For this reason, we must strengthen the cooperation between key stakeholders and ensure the implementation of scientific refinement by developing and verifying these systems.

Although water buffaloes have been neglected in all aspects compared to other farm animals, they are such animals that must be emphasized with great importance for countries whose conditions are particularly suitable with some characteristics and yields. Especially Nili-Ravi, Murrah, and Mediterranean buffalos, which are considered within the river buffalo class, may have become accustomed to machine-milking with their appropriate breast structures and high milk yields to produce up to 20–25 $\mathrm{kg}\;d^{-1}$ milk in intensive conditions (Ermetin, 2017). Water buffalo breeding, which can be realized by using PLF technologies in the field of herd management, provides benefits to breeders, animals, and consumers. However, it is only possible to obtain the desired benefits from these systems with their effective use. Unless large amounts of data obtained in many animal-related subjects are constantly used in herd management and individual decisions on animals, there will not be an advantage of intensive data flow. On the other hand, users must use the hardware and software effectively to benefit from these systems (Ermetin, 2019).

PLF technologies and practices are considered to have high potential in developing buffalo breeding around the world. In line with technological advances, with the reduction in the costs of relevant systems, PLF applications are expected to become widespread in buffalo breeding as well as in dairy cattle. As technological advances increase, costs will decrease. PLF applications are expected to become widespread in buffalo breeding, such as dairy cattle, in coming years. The individual mistakes of the shepherd and breeders are minimized in these systems, which allows them to intervene quickly in situations of possible risks.

It is possible to speculate that buffalo cultivation will become more advanced than the current position in the world and with the help of PLF technologies, increasing control and low-cost production of buffalo milk and meat products and standardization of quality. PLF technologies will increase production and quality, especially in the cultivation of intensive buffalo breeding. Scientific and technological studies must be given importance to increase the use of PLF technologies in buffalo breeding. PLF technologies must be cheap and accessible especially for small- and medium-sized businesses. It is foreseen that PLF technology applications will become widespread by reducing the costs of the systems in line with technological advances. PLF technologies used on water buffaloes (e.g., tracking systems) must be extremely resistant to environmental conditions (they must especially be waterproof).

In this respect, the development of short-, medium-, and long-term policies for education, research and support, development of practices specifically for intensive enterprises, practical training, organizing of buffalo growers, widespread research and the creation of support policies, etc. are necessary to ensure the transition to PLF technologies in buffalo breeding in the world. PLF technologies, which are used extensively in dairy cattle, must also be used in buffalo breeding. PLF technologies will be very effective in decisions for individual animals and herds, especially in selection and breeding studies.

## Data Availability

The term data in the article refers to results of survey with local breeders, and these data are given in Tables 2 and 3 provided in the body of article as well as in the statistical analysis.
